# Determinants of high mountain plant diversity in the Chilean Andes: From regional to local spatial scales

**DOI:** 10.1371/journal.pone.0200216

**Published:** 2018-07-06

**Authors:** Jesús López-Angulo, David S. Pescador, Ana M. Sánchez, Maritza A. K. Mihoč, Lohengrin A. Cavieres, Adrián Escudero

**Affiliations:** 1 Departamento de Biología, Geología, Física y Química Inorgánica, Universidad Rey Juan Carlos, Madrid, Spain; 2 Departamento de Botánica, Universidad de Concepción, Concepción, Chile; 3 Instituto de Ecología y Biodiversidad, Santiago, Chile; Chinese Academy of Forestry, CHINA

## Abstract

Mountains are considered excellent natural laboratories for studying the determinants of plant diversity at contrasting spatial scales. To gain insights into how plant diversity is structured at different spatial scales, we surveyed high mountain plant communities in the Chilean Andes where man-driven perturbations are rare. This was done along elevational gradients located at different latitudes taking into account factors that act at fine scales, including abiotic (potential solar radiation and soil quality) and biotic (species interactions) factors, and considering multiple spatial scales. Species richness, inverse of Simpson’s concentration (D_equiv_), beta-diversity and plant cover were estimated using the percentage of cover per species recorded in 34 sites in the different regions with contrasted climates. Overall, plant species richness, D_equiv_ and plant cover were lower in sites located at higher latitudes. We found a unimodal relationship between species richness and elevation and this pattern was constant independently of the regional climatic conditions. Soil quality decreased the beta-diversity among the plots in each massif and increased the richness, the D_equiv_ and cover. Segregated patterns of species co-occurrence were related to increases in richness, D_equiv_ and plant cover at finer scales. Our results showed that elevation patterns of alpine plant diversity remained constant along the regions although the mechanisms underlying these diversity patterns may differ among climatic regions. They also suggested that the patterns of plant diversity in alpine ecosystems respond to a series of factors (abiotic and biotic) that act jointly at different spatial scale determining the assemblages of local communities, but their importance can only be assessed using a multi-scale spatial approach.

## Introduction

Mountains are considered excellent natural laboratories for investigating how plant diversity is structured at contrasting spatial scales because they present extreme environmental conditions and steep climatic gradients are generated over short distances [1,2]. Spatial variation of environmental conditions varies with the grain size of investigation, and therefore, the plant community response to such abiotic heterogeneity depends on the spatial scale of observation [3–5]. Then, mountains covering an ample region and with alpine vegetation well-developed above the treeline are especially suited to infer how diversity patterns are conformed and what factors are critical at different spatial scales [6–9].

At large-scales the description of latitudinal and elevational diversity patterns have occupied a central place in ecology and most studies reveal a decreasing trend in richness with latitude and elevation [1,10–14]. Although multiple theories have been proposed in order to explain these patterns, from available space with elevation to evolutionary history [15], the intensification of environmental harshness via coldness with latitude and elevation have been frequently reported [16,17]. In any case, the relationship between diversity and elevation varies from the expected decreasing monotonic to hump-backed with richness peaks at medium elevations [13,18,19]. Such a variation could be explained by the fact that some gradients are very long and have dramatic changes in the vegetation types along them (high turnover rates along elevation within mountain ranges [18]), due to the historical human footprint and disturbances [20,21] or simply because the diversity patterns are compared at different spatial scale [9]. In other cases, the effect exerted by other concomitant regional factors may reduce the species diversity in unexpected parts of the gradient. For example, the summer drought typical of Mediterranean climate regions is exacerbated at low elevations which may interact with the opposite cold stress gradient giving unexpected patterns [22–24].

Factors operating at finer spatial scales may influence or alter those patterns found at larger spatial scales. For instance, environmental variations such as those induced by local topography (aspect or slope) at very fine scales may yield local differences in the length of the effective growing season due to different impact of solar radiation and duration of snow-free periods [25–28]. In addition, at this same fine scale, soil quality that allow greater primary productivity and plant diversity regardless of the overall habitat quality, could affect the large-scale patterns in diversity [29,30]. However, the heterogeneity produced by the differences in nutrient availability varies across scales, and thus, soil conditions may affect diversity at large scales where species are filtered from the regional species pool to small scales where plant individuals interact [31].

Superimposed, biotic interactions such as competition and facilitation are critical determinants of plant diversity at the finest spatial scales in many ecosystems [32,33], including alpine plant communities [34–37]. Specifically, the theoretical framework reveals an increase of the intensity of competition in more benign environments with a clear dominance of a reduced group of species [38], although competitive processes may promote also species diversity by niche differentiation [39]. On the other hand, when environmental conditions become more stressful, facilitative interactions become more important [40,41]. It has been shown that they can dampen the decreases in species richness acting as safety-net under harsh conditions [42]. Thus, we might not find a monotonic decrease in diversity with elevation due to local conditions.

The Chilean Andes comprise a continuous and large north to south mountain range, which leads a detailed survey of plant community diversity and determinants at multiple scales from latitude to microhabitat variation. Furthermore, in contrast to other mountain regions where the landscape and biota have been profoundly altered by human activities [43], thereby hindering the interpretation of diversity patterns [20,21], the Chilean Andes are characterized by a very low level of human-driven disturbances, especially in the southern region [44]. In addition, there are clear climate variations over a broad regional scale and it is possible to discern major differences along this mountain range. Taken all together the Chilean Andes constitutes a critical study area to gain insights into the determinants of high mountain plant diversity and to examine how plant diversity is structured at different spatial scales and if interactions between factors operating at different scales occur. Thus, we surveyed alpine plant communities along an elevational gradient at different latitudes (from –32°S to –52°S) comprising mountains with a Mediterranean-type climate where the summer drought is critical [22] to sub-Antarctic mountains where drought is negligible and summer temperature is low. Our main objectives were: (i) to determine the effect of elevation on taxonomic plant diversity (including alpha diversity as well as beta-diversity) and plant cover at different spatial scales including some contrasted latitudes along the Chilean Andes; and (ii) to determine whether factors that act at small scales, including abiotic (potential solar radiation and soil quality) and biotic factors (species interactions), might modulate the effects of latitude and elevation on different components of plant diversity. We expected taxonomic diversity to decrease with elevation conforming a monotonic pattern, but with a sharp decline in the lower elevational limit in mountains with a Mediterranean-type climate due to the effect of the summer drought at lower elevations conforming a humpbacked structure [22].

## Materials and methods

### Ethics statement

Permission for field sampling was obtained from the Gobierno de Chile and the Corporación Nacional Forestal (CONAF).

### Study area

**Fig 1:**
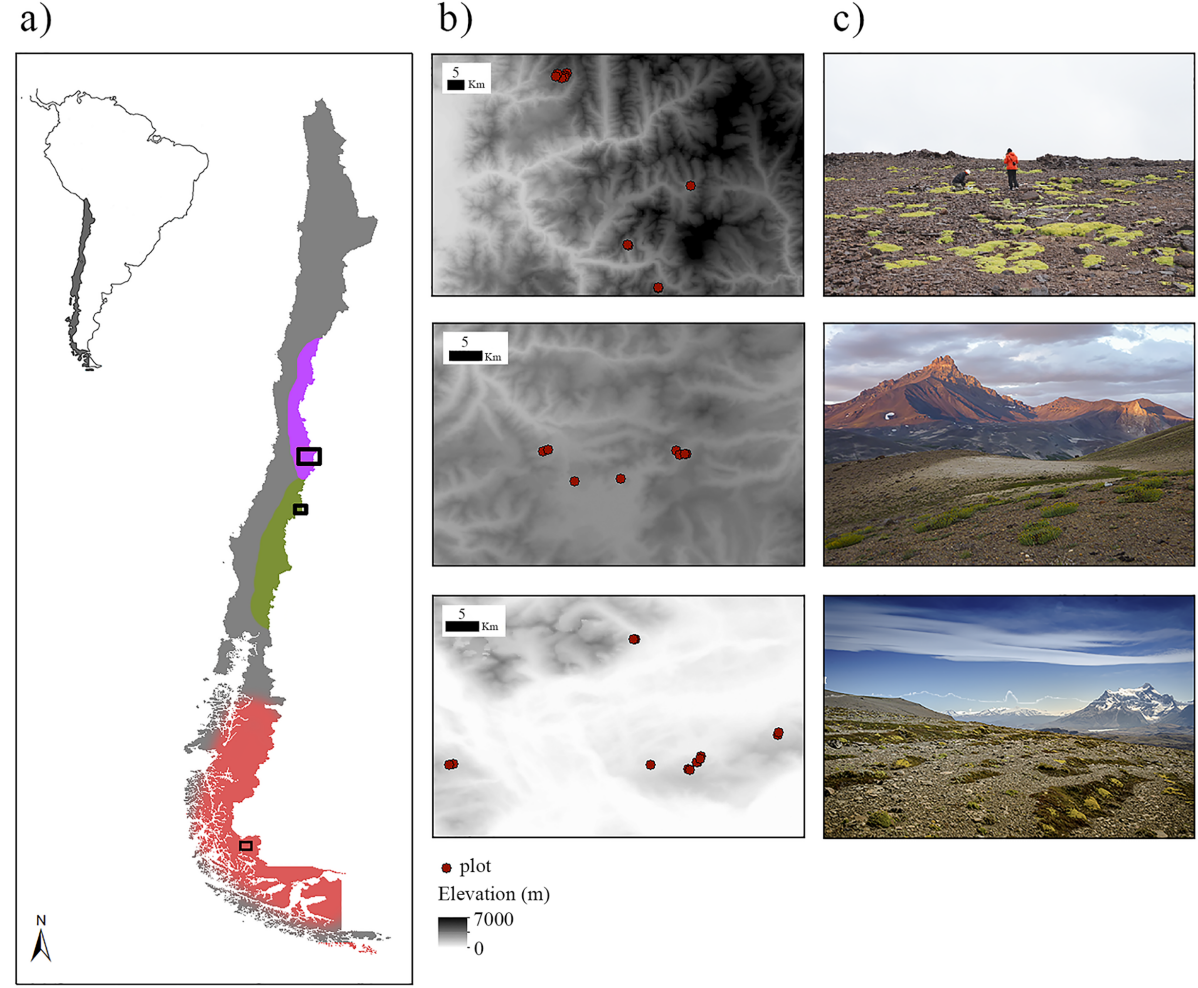
Experimental design. (a) Locations of the three study areas (black quadrats) along the Chilean Andes. Colours (purple = Mediterranean-type climate region with a severe drought summer, green = Mediterranean-type climate region with a milder drought summer and red = sub-Antarctic region) represent three different climatic zones according to Sarricolea [45]; (b) plot distribution along the three areas; and (c) typical structure of the vegetation in each area.

We selected three high mountain massifs over a long latitudinal gradient in the Chilean Andes (Fig 1A and 1B): (1) Farellones (33°2′S, 70°1′W) located 40 km east of the city of Santiago; (2) Maule (35°6′S, 70°3′W) situated 100 km east of the city of Talca; and (3) Torres del Paine National Park (51°0′S, 73°0′W) hereafter referred to as Torres del Paine, and located in sub-Antarctic Andes. Farellones and Maule are influenced by a Mediterranean-type climate but the length of the summer drought is significantly longer at Farellones than at Maule [45] and Torres del Paine has a sub-Antarctic climate without summer drought and rainfall distributed evenly throughout the year [46].

In Farellones, where treeline is situated at 2200 m and the dominant treeline species is *Kageneckia angustifolia* D. Don (Rosaceae), the mean annual temperature and precipitation are 6.5°C and 943 mm, respectively [47,48]. In Maule where the treeline is situated at 1700 m, the dominant treeline species is *Nothofagus antarctica* (G. Forst.) Oerst (Nothofagaceae) accompanied by *Austrocedrus chilensis* (D.Don) Pic-Serm. & Bizzarri (Cupressaceae). The mean annual temperature and precipitation at this location are 5°C and 900 mm [49], respectively. In Torres del Paine, the treeline is situated at 500 m and is formed by *Nothofagus pumilio* (Poepp & Endl.) Krasser (Nothofagaceae). The mean annual temperature at this location is 5°C and annual precipitation varies around 900–1000 mm [46].

The vegetation above the treeline (Fig 1C) is generally dominated by caespitose herbs (e.g. *Poa denudata* Steud), which are accompanied by other growth forms such as prostrate shrubs (e.g., *Berberis empetrifolia* Lam.), perennial forbs (e.g., *Phacelia secunda* J.F. Gmel. and *Nassauvia pyramidalis* Meyen), and cushion-like plants (e.g., *Azorella madreporica* Clos and *Laretia acaulis* (Cav.) Gillies & Hook).

### Field sampling

Field sampling was performed in the Mediterranean and sub-Antarctic climate regions during the summer in 2014 and 2015, respectively, in the summer season when the alpine plants were at their phenological peak. We sampled a total of 34 sites (11 in Farellones, 9 in Maule, and 14 in Torres del Paine) above the treeline selecting good representatives of alpine vegetation avoiding, rocks, screes, snow beds and disturbed areas, and covering the complete elevational range where the ecosystem occurs in each region. The sampled elevation ranged from 2477 to 3627 in Farellones, from 2064 to 2666 in Maule, and from 500 to 1050 m in Torres del Paine. In addition to the variation between regions and the complete elevational range taken in each mountain massif, at each site, the community structure was surveyed at three different spatial scales: (1) plot scale: one 20 m × 20 m sampling plot per site attending to the macroclimate (via elevation) and microclimate (via slope and orientation); (2) quadrat scale: five 2.4 m × 2.4 m quadrats were established within each plot, with one in each corner and a fifth in the centre corresponding to the scale in which in these communities the microsite variability (via soil heterogeneity) is better expressed; and (3) cell scale: the central quadrat was divided into 64 cells measuring 30 cm × 30 cm, with a total of 2176 cells representing the scale in which plant to plant variations are critical. The percentage cover of each species was visually estimated in each quadrat and in each cell. The plant cover per plot was calculated indirectly as the sum of the mean cover of each species in the five quadrats, where the plant cover in the central quadrat was estimated as the mean cover by each species in the 64 cells.

The cell data information (the percentage cover of each species in the 64 grids of 30 cm x 30 cm in each site) obtained at the finest scale was used to estimate a checkerboard score (c-score [50])which quantifies the degree of spatial segregation for species co-occurrence, as an integrative estimate of plant to plant interactions. High c-score values denote that species pairs occur less frequently whereas low values indicate a tendency for species to aggregate spatially. The average size of the plant species in the community was around 15 cm in diameter, so the cell size appeared to be adequate for estimating real biotic interactions.

At each site (plot scale) we measured the elevation and slope aspect using a GPS (Garmin Colorado-300, Garmin Ltd, Olathe, USA), and a clinometer for the slope (Silva Clinomaster, Silva Sweden, Sollentuna, Sweden). Elevation was standardized among the different mountain massifs in order to compare regions at various latitudes by subtracting the mean treeline elevation (determined using Google Earth images) from the plot elevation. Aspect and slope values were used to calculate Gandullo’s potential solar radiation coefficient (for details see [24,51]). Mean annual temperature and annual precipitation were extracted from the Worldclim database (www.worldclim.org, resolution 30 [52]).

In addition, at the quadrat scale we evaluated the soil quality by taking two soil cores with a diameter of 5 cm and depth of 10 cm from each corner-quadrat, with one from an open area and the other under the perennial and dominant plant species. This yielded eight soil samples per plot. The soil samples were air dried for one month and then sieved through a 2-mm mesh. We assessed eight multi-functional ecosystem properties related to the cycling and storage of nutrients. In particular, we selected organic carbon (C), total nitrogen (N), available phosphorus (P), and potassium (K) as key nutrients related to primary productivity and the buildup of nutrient pools [53]. These parameters are also surrogates for other forms of C, N, and P that are available to plants [54] and they can be treated as ecosystem functions related to soil fertility and primary productivity. Furthermore, we estimated the enzymatic activities of phosphatase and β-glucosidase, which are closely related to the microbial functionality and nutrient dynamics in soil. Soil organic C was determined by colorimetry after oxidation with a mixture of potassium dichromate and sulfuric acid [55]. Total N and available P were determined with a SKALAR++ San Analyzer (Skalar, Breda, The Netherlands) in our laboratory after digestion with sulfuric acid and Kjedahl’s catalyst [56]. Potassium (K) was measured with the same analyzer system after shaking the soil samples with distilled water (1:5 ratio) for 1 h. Enzymatic activities were estimated using the methods described by Eivazi and Tabatabai (β-glucosidase [57]) and Tabatabai and Bremner (acid phosphatase [58]). The soil pH and electrical conductivity were measured in a soil and water suspension at a mass:volume ratio of 1:3 using a pH meter (GLP 21; Crison, Barcelona, Spain) and a conductivity meter (GLP 31; Crison, Barcelona, Spain), respectively. These variables were then averaged to obtain quadrat-level estimates based on the mean values determined in bare ground and vegetated areas, where they were weighted by the respective cover value in each quadrat. The centre quadrat value was estimated as the average of the four quadrats at each site. All of these soil variables are determinants of the functioning of ecosystems [59,60], so we calculated an ecosystem multi-functionality index [53]:

$$M_{i}=\left(\sum_{k=1}^{n}\left(x_{ki}-\mu_{k}\right)/\sigma_{k}\right)/n,$$ (1)

where *M*_*i*_ is the multi-functionality of plot *i*, *n* is the total number of soil parameters, *x*_*ki*_ is the value of parameter *k* in plot *i*, and μ_*k*_ and σ_*k*_ are the mean and standard deviation for each parameter *k*, respectively.

Note that for elevation, potential solar radiation and c-score we have a unique value for all scales, whereas that the soil quality values were particular for each quadrat and a unique value for each plot and all cells.

### Diversity metrics

Species richness (S) was estimated as the number of plant species recorded in each sampling unit (i.e., cells, quadrats and plots). We estimated the inverse of Simpson’s concentration index (D_equiv_) expressed as species richness equivalents as an additional alpha diversity measure according to Jost [61]:

$$D_{equiv}=1/\sum_{i}^{S}p_{i}^{2},$$ (2)

where *p*_*i*_ is the cover proportion of species i and S is species richness. Cover data were square root-transformed before estimating D_equiv_ and the beta-diversity. We also measured beta-diversity to assess the non-directional variation in species composition across sampling units [62]. It was calculated as the mean pairwise Bray–Curtis floristic dissimilarities among samples (i.e., cells, quadrats and plots) within each group depending on the spatial scale [62]:

$$\bar{d}=\frac{1}{n-1}\sum_{i,j<i}d_{ij},$$ (3)

where n is the number of samples within each group and d_ij_ is the dissimilarity of a target sample *i* relative to another sample *j*. Thus, *plot beta-diversity* was the mean of all the pairwise dissimilarities between a target plot and the other plots within each of the three mountainous massifs. *Quadrat beta-diversity* was the mean of all the pairwise dissimilarities between a target quadrat and the other four quadrats within each of the 34 plots. *Cell beta-diversity* was the mean of all the pairwise dissimilarities between a target cell and the other 63 cells within each of the 34 centre quadrats. Finally, we measured plant cover (C, estimated as the sum of the cover by all species because the level of overlapping was very low in these plant communities) in each sampling unit as a surrogate for productivity.

### Statistical analyses

The standardized elevation was highly correlated to mean annual temperature (r^2^ = 0.9) and annual precipitation (r^2^ = 0.6), so theses climatic variables were excluded from further analyses because standardized elevation is more reliable. The relationships between the standardized elevation, soil quality assessed by soil multi-functionality, potential solar radiation, biotic interactions (c-score), and the diversity metrics were analysed using generalized linear models (GLMs) at the plot scale, and with generalized linear mixed-effects models (GLMMs) at the quadrat and cell scales. The response of species richness to the predictors was evaluated with a Poisson error distribution and logarithmic link function, and the response of D_equiv_, beta-diversity and total plant cover were analysed using a Gaussian error distribution and identity link function.

We analysed the variation of the diversity metrics between the different regions (i.e., Farellones, Maule, and Torres del Paine) by including this factor as a fixed factor. Post hoc Tukey tests were performed to detect any significant differences among regions. We also included plot as a random factor. The convenience of including the quadratic term of elevation and the interaction between elevation and massifs in the final models was evaluated using the AICc criterion. We checked for collinearity between the different environmental predictors using the variance inflation factor before implementing the models where they were below 2 in all cases, thereby indicating the absence of problems with co-linearity [63]. The normality of the standardized residuals was confirmed visually for all of the models. We square root-transformed the total plant cover in order to normalize the data before conducting the analyses. We estimated the statistical significance of each predictor using type-II analysis of variance. We calculated the total variance explained (R^2^) by each GLM, and the conditional variance explained (R^2^_c_) by both fixed and random factors, as well as the marginal variance explained (R^2^_m_) by fixed factors for each GLMM using the MuMIn package. All of the statistical analyses were performed in R (v 3.2.4) using the lme4, car, and vegan packages [64].

## Results

We recorded a total of 234 perennial plant species (a list of taxa are provided in S1 Table) in the 34 plots sampled in the three regions, with a total of 86, 86, and 118 species in the Farellones, Maule, and Torres del Paine, respectively. Richness ranged from 12 to 50 species per plot, with a mean of 25.3 (± 10) plant species per plot (other mean of diversity metrics on three regions at three scales are provided in S2 Table). The most abundant species in Farellones were the cushion plants *Azorella madreporica* and *Laretia acaulis*, and graminoids such as *Rytidosperma pictum* and *Poa cf*. *denudata*. The graminoids comprising *Festuca acanthophylla* and *Poa* cf. *denudata* were dominant in the Maule region. Finally, in the sub-Antarctic region, the community was dominated by prostrate shrubs such as *Empetrum rubrum* at low elevations, whereas the higher zones were dominated by the cushion plant *Azorella monantha*.

The fitted GLMs and GLMMs explained a high proportion of the variance in the diversity component at all of the spatial scales considered (Table 1). There were significant relationships between all of the diversity metrics (i.e., species richness, D_equiv_, beta-diversity, and total plant cover) and some of the considered predictors.

**Table 1:** Coefficients of the models (GLMs and GLMMs) examining the effects of environmental factors on the diversity indices at plot (20 x 20 m), at quadrat (2.4 x 2.4 m) and cell (30 x 30 cm) scales.

		plot scale		quadrat scale			cell scale		
Species richness									
	Intercept (Farellones)	3.08		2.27			0.48		
	Massif		**		**			**	
	Maule	0.24	ab	0.26	ab		0.63	ab	
	Torres del Paine	0.29	b	0.43	b		0.63	b	
	Elevation	-0.12	**	-0.08			-0.02		
	Elevation^2^	-0.10	**						
	Soil quality	0.20	***	0.14	**		0.32	**	
	Potential solar radiation	0.02		0.04			0.07		
	C-score	0.07		0.19	***		0.45	***	
	R^2^	0.89							
	R^2^m			0.39			0.41		
	R^2^c			0.70			0.56		
Inverse of Simpson’s concentration									
	Intercept (Farellones)	6.21		4.91			1.20		
	Massif		**		**			***	
	Maule	9.50	b	3.47	ab		1.55	b	
	Torres del Paine	9.12	b	4.62	b		1.52	b	
	Elevation	-0.16		0.06			0.17		
	Soil quality	3.80	*	0.99	*		0.69	**	
	Potential solar radiation	0.07		0.29			0.03		
	C-score	1.32		1.88	**		1.01	***	
	R^2^	0.50							
	R^2^m			0.32			0.38		
	R^2^c			0.67			0.53		
Beta-diversity									
	Intercept (Farellones)	0.86		0.48			0.78		
	Massif		***						
	Maule	-0.05	a	-0.01			0.04		
	Torres del Paine	-0.16	b	0.05			0.01		
	Elevation	-0.04	**	0.03			0.00		
	Soil quality	-0.04	*	-0.00			-0.01		
	Potential solar radiation	0.01		-0.01			-0.03		
	C-score	-0.01		0.03			-0.06		
	R^2^	0.67							
	R^2^m			0.18			0.16		
	R^2^c			0.64			0.63		
Plant cover									
	Intercept (Farellones)	14.134		2.634			2.16		
	Massif		*						
	Maule	7.599	ab	-0.065			0.17		
	Torres del Paine	15.836	b	0.242			-0.07		
	Elevation	-2.825	*	-0.239	*		0.03		
	Soil quality	9.081	**	0.286	*		0.43	*	
	Potential solar radiation	1.649		0.051			0.20		
	C-score	2.849		0.197	*		0.32	*	
	R^2^	0.62							
	R^2^m			0.25			0.17		
	R^2^c			0.39			0.33		

Elevation^2^: the quadratic term of Elevation.

R^2^: variance explained by each model. R^2^m: marginal variance explained by fixed factors. R^2^c: conditional variance explained by both fixed and random factors.

The significance is shown as

*** P<0.001

** P<0.005

* P<0.05.

Different letters within columns indicate significant differences (P< 0.05) between Farellones (a) and the other two regions according to Tukey's range test

### Effects of latitude

Species richness, D_equiv_ and total plant cover varied significantly among regions at the plot scale (Table 1). Species richness and total plant cover were significantly higher in the Torres del Paine than in Farellones and D_equiv_ was significantly lower in Farellones than in the other two sites (Table 1). The differences in species richness and D_equiv_ among regions were consistent across the three spatial scales (Table 1). Contrarily we only found significant differences of total plant cover among regions only at the plot scale. The dissimilarity in species composition (βeta-diversity) among plots in each massif was significantly lower in Torres del Paine than in Farellones (Table 1).

### Effects of elevation

At the plot scale, the quadratic relationship between elevation and species richness significantly improved the goodness of fit (AICc with quadratic term = 246.2 vs. without = 251.6). In addition, a more complex model including the interaction between elevation and massifs produced lower goodness of fit for all diversity metrics and scales. The negative quadratic relationship between elevation and species richness (Table 1) indicated that the number of species was higher toward intermediate elevations above the local treeline (500–700 m standardized elevation) with a decrease toward both edges, which was more pronounced toward the upper limit. This pattern was observed across the different regions despite species richness differed among them. D_equiv_ showed no statistically significant relationship with elevation. Lastly, the beta-diversity exhibited a strong monotonic decrease with elevation at the plot scale but not at smaller ones (Table 1).

### Effects of local abiotic environment

We found a positive correlation between soil quality with species richness, the D_equiv_ and total plant cover. These relationships were maintained across the three spatial scales (Table 1). The beta-diversity decreased as soil quality increased at the plot scale (Table 1). There were no significant relationships between the diversity metrics and solar potential solar radiation (Table 1).

### Effects of species interactions

The c-score index was positively associated with all the diversity metrics at fine spatial scales. Species richness, D_equiv_, and plant cover increased as the frequency of species co-occurrence decreased at the quadrat and cell scales. Thus, a shift from an aggregated to segregated species co-occurrence pattern was observed with the increase in richness, D_equiv_ and plant cover (Table 1). In addition, species segregation was associated with decreases in the mean pairwise dissimilarities (beta-diversity) between cells.

## Discussion

Our findings showed that the patterns of plant diversity in alpine ecosystems as hypothesized respond to a series of factors that act at different spatial scale, i.e. climatic variation related to latitude, local variations in elevation and fine scale species interactions. Our results support the idea that the general variation in plant diversity with latitude and elevation, questions usually tackled by biogeographers and macroecologists [11,12,65], can be modified by the effect exerted by other concomitant factors acting at smaller scales [13,18–21].

Most empirical diversity studies have shown the well-documented latitudinal pattern of plant species diversity decline with latitude which is mostly constrained by solar energy inputs towards the poles [11,12,66]. By contrast, our findings across latitude in the high Chilean Andes showed an opposite pattern of decreasing plant diversity and total plant cover (as surrogate of primary productivity) as the distance from the Equator decreased. As hypothesized, this decline in diversity seems to be related with the existence of a regional gradient generated by the intense summer drought whose effect is diluted from Mediterranean type climate to the sub-Antarctic regions where water stress is practically absent and soil water is available throughout the growing season. Although some authors have suggested that water deficit is not a critical determinant of plant diversity in alpine habitats [1] our results concurs with others in temperate mountains [67–69], and particularly in Mediterranean regions [22,23,70].

We found that species richness exhibited a unimodal relationship with the standardized elevation, with the maximum values at medium elevations within regions and the minimum values at both edges of the gradient. Surprisingly, this pattern was similar along the three massifs independently of the regional climatic conditions. The expected pronounced decrease in richness (and total plant cover) with elevation is due to the environmental severity [13,18], which increases with elevation because of coldness, short growing seasons, excessive radiation, and other factors [1]. This would induce a monotonic relationship, which is then modulated by other local factors to obtain a unimodal pattern. In our opinion, the factors responsible for generating this humped pattern differed among climates. For instance, high mountain Mediterranean-type massifs are characterized by summer drought at low elevations, which shortens the growing season [22]. This implies that stress is intense at both ends of the elevational gradient with coldness at the highest elevations and summer drought at the lowest elevations, but more benign conditions at intermediate elevations [71] and therefore greater species richness. As commented some previous studies have suggested that water deficit is not a critical determinant of plant diversity in alpine habitats [1] but our results are in accordance with others obtained in Mediterranean regions [22,23,70].

**Fig 2:**
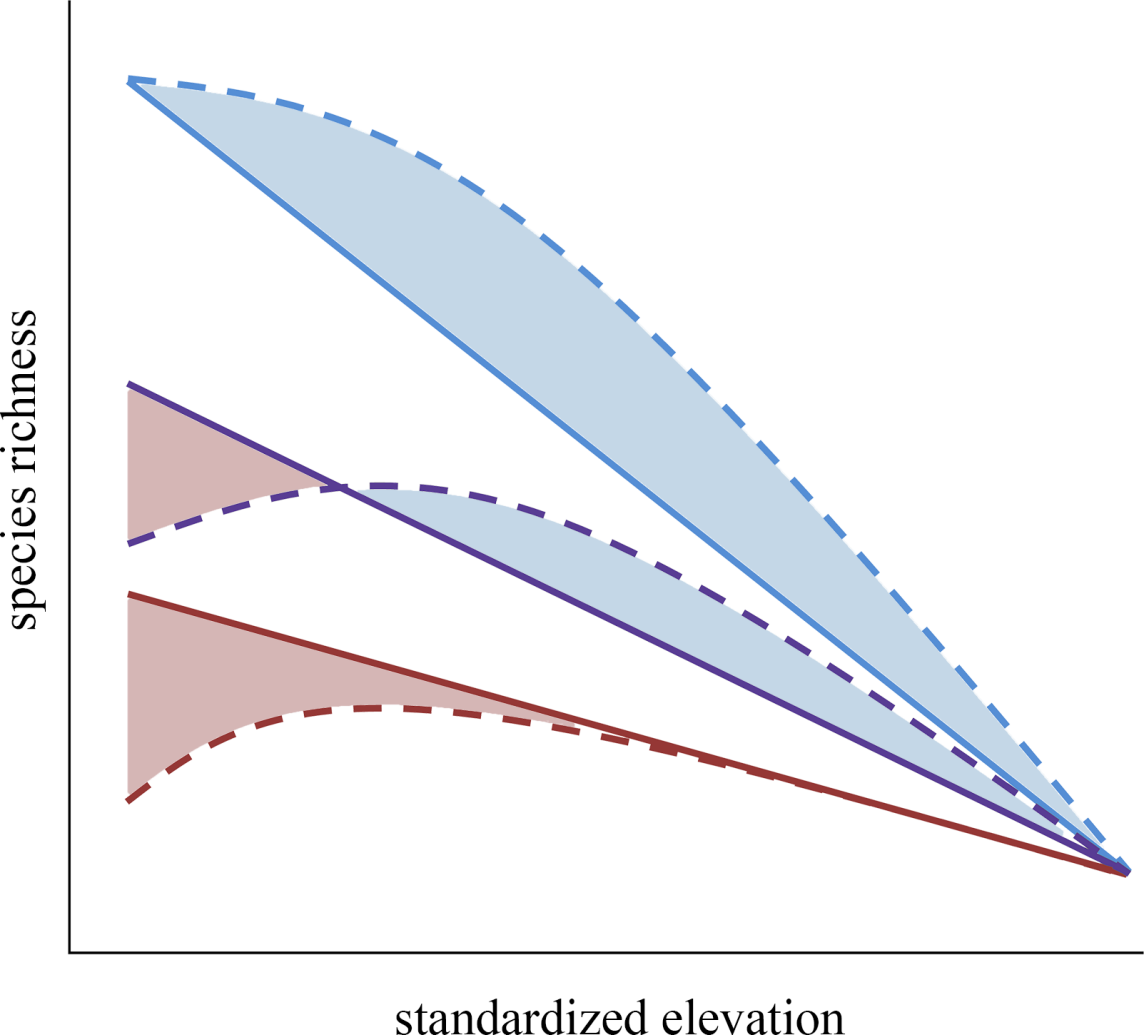
Conceptual diagram showing the relationship between species richness and the standardized elevation across contrasted latitudes. Species richness variation along an elevational gradient in a sub-Antarctic mountain (blue lines) and in two Mediterranean-climate type mountains with different length in dry season: long dry season (red lines) and short dry season (purple lines). The solid lines represent the richness patterns when the main environmental stressor is coldness. The dotted lines represent the richness patterns when summer drought (red dotted line), facilitation (blue dotted line), or both mechanisms (purple dotted lines) act modulating the original monotonic pattern. Decreasing and increasing species richness are represented by the red and blue shaded area, respectively.

In the temperate sub-Antarctic Chilean Andes, summer drought is practically negligible, thus the humped pattern in richness along elevation may be related to other mechanisms. The mass effect in an ecotone zone [18], and the higher intensity of competitive exclusion [25,38] and disturbance (e.g. grazing [72]) are factors that have been claimed to explain this pattern in other sub-Arctic mountains. However, we suggest this diversity pattern in Patagonian Andes is attributable to the effect and prevalence of positive plant–plant interactions at intermediate elevations. It is known that facilitation by cushion plants and other nurse plants that dominate alpine ecosystems increases species richness at the entire community level [37]. Many studies have also shown that the magnitude of facilitation increases with the stress level (i.e., elevation), which could led to an increase in richness as elevation increases [40,46,73]. Nonetheless, the intensity of the facilitative effect declines under extremely stressful conditions [41], and thus a decay in species richness is expected at the uppermost limit of the elevational gradient due to coldness. If so, processes acting at very fine scale such as biotic interactions may modulate the effect of elevation on plant diversity. Our results suggest that different mechanisms can probably generate a very similar humpback pattern under contrasting climates and independently of the net differences in species richness along the Andes (Fig 2).

More direct evidences of the importance of biotic interactions in the structuration of diversity in these mountains were supported by the significant relation with plant diversity and also with productivity (i.e., total plant cover) at the quadrat and cell scales. We found that species segregation was related to higher richness, D_equiv_, and total plant cover, thereby suggesting niche differences and spatial repulsion of species to avoid or reduce competition [74–76], which allowed more species to co-occur. However, the species co-occurrence patterns were not related to any of the plant diversity metrics at the plot scale. Therefore, the effects of species interactions could only be detected at the spatial scale where individuals could potentially interact. However, it is worth mentioning that c-score is calculated as the mean average pairwise co-occurrence of all species, both the benefactor and beneficiary species, and thus, the expected facilitative effect that the benefactor nurses produce could become blurred.

An increase in harshness with elevation could be related to the parallel decrease in beta-diversity which reflects species compositions of the assemblages more similar as elevation increased within each region. Other studies performed along elevational gradients also found a decrease in beta-diversity toward high elevations [77–79]. Our results suggest the existence and prevalence of abiotic filters with elevation, thereby reducing the available species pool and leading to more homogeneous plant assemblages. In addition, soil quality that relates to critical ecosystem functions, such as carbon storage, productivity, and the build-up of nutrient pools [53], decreased the composition dissimilarity among plots in each massif. The differences in beta-diversity among communities may be related to the dominance of prostrate nurses and cushion-like shrubs such as *Azorella madreporica* and *Empetrum rubrum*. These species enhance soil quality because they increase the availability of nutrients under their canopies [71,80,81] generating fertility islands and increments in primary productivity [81,82]. This implies that nurse species produce improvements of soil quality and amelioration of the extreme environmental conditions generating more stable and predictable conditions compared to the surrounding environment areas leading to species rich and constant assemblages, resulting again in a decrease in beta-diversity among plots [83].

Our results showed that the patterns of taxonomic diversity in alpine ecosystems are related to both large-scale variables (climate estimated indirectly based on elevation and latitude) and small-scale variables (soil quality and biotic interactions), which jointly determine the assemblages of local communities and the patterning of diversity as a whole. Our findings demonstrate that a multi-scale approach is necessary to elucidate the mechanisms that shape alpine plant diversity over a large area because the effect of abiotic and biotic factors appeared to be patent only at particular spatial scales. For example, elevation influenced the total number of species and plant cover, and these effects were clearly detectable at the plot scale, whereas the c-score affected diversity only at the finest scales (quadrat and cell). In addition, our results demonstrated that the patterns of taxonomic diversity with elevation remain constant along the regions in the Andes, although the mechanisms responsible for causing and maintaining these patterns differ among regions. The summer drought has important effects on the Andean communities in the central Mediterranean-type climate region of Chile [23,84] whereas facilitation may be critical in other regions. The combined effects of local biotic processes (such as facilitation) acting over large-scale abiotic gradients as well as regional factors determine the community assembly and the overall diversity patterns in stressed ecosystems.

## Supporting information

S1 Table List of plant species occurring at each region. (PDF)

S2 Table The mean (± standard error) of all metrics of taxonomic community structure on three regions at plot, quadrat and cell scales. (PDF)
