# Supplementary material for: Determinants of high mountain plant diversity in the Chilean Andes: From regional to local spatial scales
Source: PLoS One. 2018 Jul 6;13(7):e0200216. doi: 10.1371/journal.pone.0200216 (PMC6034847; doi:10.1371/journal.pone.0200216)
Supplement: S1 Table — (PDF) [file pone.0200216.s001.pdf]

S1 Table: List of plant species occurring at each region

Family	Specie	Farellones	Maule	Torres del Paine
Rosaceae	*Acaena antarctica* Hook. f.			X
Rosaceae	*Acaena leptacantha* Phil.		X	
Rosaceae	*Acaena lucida* (Aiton) Vahl			X
Rosaceae	*Acaena macrocephala* Poepp.		X	
Rosaceae	*Acaena magellanica* (Lam.) Vahl			X
Rosaceae	*Acaena pinnatifida* Ruiz & Pav.	X	X	X
Rosaceae	*Acaena sericea* J. Jacq.			X
Rosaceae	*Acaena* sp.			X
Rosaceae	*Acaena splendens* Hook. & Arn.	X		
Fabaceae	*Adesmia boronioides* Hook. f.		X	
Fabaceae	*Adesmia capitellata* (Clos) Hauman	X		
Fabaceae	*Adesmia corymbosa* Clos			X
Fabaceae	*Adesmia glomerula* Clos	X	X	
Fabaceae	*Adesmia longiseta* DC.	X		
Fabaceae	*Adesmia lotoides* Hook. f.		X	X
Fabaceae	*Adesmia montana* Phil.	X		
Fabaceae	*Adesmia pumila* Hook. f.			X
Fabaceae	*Adesmia sandwithii* Burkart			X
Fabaceae	*Adesmia villosa* Hook. f.			X
Poaceae	*Agrostis* sp. 1			X
Poaceae	*Agrostis* sp. 2			X
Alstroemeriaceae	*Alstroemeria pallida* Graham	X		
Alstroemeriaceae	*Alstroemeria umbellata* Meyen	X		
Brassicaceae	*Alyssum alyssoides* L.			X
Fabaceae	*Anarthrophyllum desideratum* (DC.) Benth.			X
Ranunculaceae	*Anemone multifida* Poir.			X
Caryophyllaceae	*Arenaria serpens* Kunth		X	
Schoepfiaceae	*Arjona patagonica* Hombr. & Jacq. ex Decne			X
Plumbaginaceae	*Armeria maritima* (Mill.) Willd.		X	X
Asteraceae	*Asteraceae* sp.			X
Fabaceae	*Astragalus cruckshanksii* (Hook. & Arn.) Griseb.	X		
Fabaceae	*Astragalus palenae* (Phil.) Reiche			X
Fabaceae	*Astragalus* sp.	X		
Poaceae	*Avenella flexuosa* (L.) Drejer			X
Apiaceae	*Azorella filamentosa* Lam.			X
Apiaceae	*Azorella lycopodioides* Gaudich.			X
Apiaceae	*Azorella madreporica* Clos	X		
Apiaceae	*Azorella monantha* Clos		X	X
Apiaceae	*Azorella selago* Hook. f.			X
Apiaceae	*Azorella trifurcata* (Gaertn.) Pers.			X
Asteraceae	*Baccharis magellanica* (Lam.) Pers.			X
Asteraceae	*Baccharis neaei* DC.		X	

Ranunculaceae	*Barneoudia major* Phil.	X		X
Asteraceae	*Belloa chilensis* (Hook. & Arn.) J. Remy		X	
Berberidaceae	*Berberis empetrifolia* Lam.	X	X	X
Berberidaceae	*Berberis microphylla* G. Forst.			X
Blechnaceae	*Blechnum penna-marina (*Poir.) Kuhn			X
Apiaceae	*Bolax caespitosa* Hombr. & Jacq. ex Decne.			X
Apiaceae	*Bolax gummifera* (Lam.) Spreng.			X
Poaceae	*Bromus catharticus* Vahl	X		
Poaceae	*Bromus setifolius* J. Presl	X	X	X
Poaceae	*Bromus* sp.		X	
Montiaceae	*Calandrinia compacta* Barnéoud		X	
Calceolariaceae	*Calceolaria arachnoidea* Graham	X		
Calceolariaceae	*Calceolaria biflora* Lam.			X
Calceolariaceae	*Calceolaria corymbosa* Ruiz & Pav.	X		
Calceolariaceae	*Calceolaria filicaulis* Clos		X	
Calceolariaceae	*Calceolaria polyrrhiza* Cav.		X	
Calceolariaceae	*Calceolaria uniflora* Lam.			X
Cyperaceae	*Carex andina* Phil	X	X	
Cyperaceae	*Carex aphylla* Kunth		X	
Cyperaceae	*Carex atropicta* Steud.		X	
Caryophyllaceae	*Cerastium arvense* L.	X	X	X
Asteraceae	*Chaetanthera euphrasioides* (DC.) F. Meigen	X	X	
Asteraceae	*Chaetanthera lycopodioides* (Remy) Cabrera ex Cabrera			
Dennstaedtiacea	*Cheilanthes glauca* (Cav.) Mett.	X		
Orchidaceae	*Chloraea magellanica* Hook. f.			
Asteraceae	*Chuquiraga oppositifolia* D. Don	X		
Montiaceae	*Cistanthe picta* (Gillies ex Arn.) Carolin ex Hershkovitz		X	
Montiaceae	*Cistanthe* sp.	X		
Lamiaceae	*Clinopodium darwinii* (Benth.) Kuntze			X
Caryophyllaceae	*Colobanthus lycopodioides* Griseb.			X
Convolvulaceae	*Convolvulus demissus* Choisy		X	
Apiaceae	*Daucus montanus* Humb. & Bonpl. ex Spreng.			X
Apocynaceae	*Diplolepis nummulariifolia* (Hook. & Arn.) Liede & Rapini	X		
Rhamnaceae	*Discaria chacaye* (G. Don) Tortosa	X		
Brassicaceae	*Draba funiculosa* Hook. f.			X
Brassicaceae	*Draba gilliesii* Hook. & Arn.	X		
Brassicaceae	*Draba magellanica* Lam.			X
Poaceae	*Elymus magellanicus* (E. Desv.) A. Löve			X
Empetraceae	*Empetrum rubrum* Vahl ex Willd.			X
Ephedraceae	*Ephedra chilensi*s C. Presl		X	X
Ephedraceae	*Ephedra frustillata* Miers			X
Asteraceae	*Erigeron andicola* DC.	X	X	X
Asteraceae	*Erigeron leptopetalus* Phil.		X	X
Asteraceae	*Erigeron myosotis* Pers.		X	X
Escalloniaceae	*Escallonia rubra* (Ruiz & Pav.) Pers.			X
Euphorbiaceae	*Euphorbia collina* Phil.	X	X	X

Poaceae	*Festuca acanthophylla* E. Desv.		X	
Poaceae	*Festuca gracillima* Hook. f.			X
Poaceae	*Festuca magellanica* Lam.		X	
Poaceae	*Festuca pyrogea* Speg.			X
Poaceae	*Festuca* sp.		X	
Rubiaceae	*Galium antarcticum* Hook. f.			X
Rubiaceae	*Galium eriocarpum* Bartl. ex DC.		X	
Asteraceae	*Gamochaeta nivalis* Cabrera			X
Asteraceae	*Gamochaeta* sp.		X	
Ericaceae	*Gaultheria caespitosa* Poepp. & Endl.			X
Geraniaceae	*Geranium sessiliflorum* Cav.	X		X
Gunneraceae	*Gunnera magellanica* Lam.			
Ranunculaceae	*Hamadryas delfinii* Phil. ex Reiche			X
Asteraceae	*Haplopappus anthylloides* Meyen & Walp.	X	X	
Poaceae	*Hordeum comosum* J. Presl	X	X	X
Asteraceae	*Hypochaeris clarionoides* (J. Remy) Reiche	X		
Asteraceae	*Hypochaeris incana* (Hook. & Arn.) Macloskie			X
Asteraceae	*Hypochaeris tenerifolia* (J. Remy) Dusén			X
Juncaceae	*Juncaceae* sp.			X
Juncaceae	*Juncus balticus* Willd.		X	
Verbenaceae	*Junellia tridens* (Lag.) Moldenke			
Apiaceae	*Laretia acaulis* (Cav.) Gillies & Hook.	X	X	
Liliaceae	*Latace andina* (Poepp.) Sassone	X		
Fabaceae	*Lathyrus magellanicus* Lam.			X
Asteraceae	*Leucheria leontopodioides* (Kuntze) K. Schum.	X		X
Asteraceae	*Leucheria lithospermifolia* (Less.) Reiche		X	
Asteraceae	*Leucheria millefolium* Dusén & Skottsb.		X	
Asteraceae	*Leucheria senecioides* Hook. & Arn.	X		
Asteraceae	*Leucheria viscida* (Bertero ex Colla) Crisci	X		
Loasaceae	*Loasa pinnatifida* Gillies ex Arn.	X		
Loasaceae	*Loasa sigmoidea* Urb. & Gilg	X	X	
Juncaceae	*Luzula alopecurus* Desv.	X	X	X
Juncaceae	*Luzula parvula* Barros	X	X	
Juncaceae	*Luzula racemosa* Desv.		X	
Juncaceae	*Luzula* sp.			X
Juncaceae	*Marsippospermum reichei* Buchenau			X
Montiaceae	*Melosperma andicola* (Gillies) Benth.	X		
Brassicaceae	*Menonvillea cuneata* (Gillies & Hook.) Rollins		X	
Brassicaceae	*Menonvillea scapigera* (Phil.) Rollins	X		
Brassicaceae	*Microsteris gracilis* (Hook.) GreenePolemoniaceae	X		
Montiaceae	*Montiopsis andicola* (Gillies) D.I. Ford	X		
Montiaceae	*Montiopsis gayana* (Barnéoud) D.I.Ford		X	
Montiaceae	*Montiopsis potentilloides* (Barnéoud) D.I. Ford	X		
Montiaceae	*Montiopsis* sp.		X	
Montiaceae	*Montiopsis potentilloides* (Barnéoud) D.I. Ford	X		
Apiaceae	*Mulinum spinosum* (Cav.) Pers.		X	X

Asteraceae	*Mutisia linearifolia* Cav.	X		
Asteraceae	*Mutisia sinuata* Cav.	X		
Boraginaceae	*Myosotis* sp.			X
Asteraceae	*Nardophyllum bryoides* (Lam.) Cabrera			X
Asteraceae	*Nassauvia aculeata* (Less.) Poepp. & Endl.			X
Asteraceae	*Nassauvia darwinii* (Hook. & Arn.) O. Hoffm. & Dusén			X
Asteraceae	*Nassauvia digitata* Wedd.		X	
Asteraceae	*Nassauvia dusenii* O. Hoffm.			X
Asteraceae	*Nassauvia glomerata* (Gillies ex D. Don) Wedd.	X	X	
Asteraceae	*Nassauvia lagascae* (D. Don) F. Meigen		X	
Asteraceae	*Nassauvia maeviae* Cabrera			X
Asteraceae	*Nassauvia pygmaea* Hook.f.			X
Asteraceae	*Nassauvia pyramidalis* Meyen		X	
Asteraceae	*Nassauvia revoluta* D. Don	X	X	
Calyceraceae	*Nastanthus scapigerus* (J. Remy) Miers	X	X	
Calyceraceae	*Nastanthus ventosus* (Meyen) Miers	X	X	X
Brassicaceae	*Noccaea magellanica* (Comm. ex Poir.) Holub	X	X	
Nothofagaceae	*Nothofagus pumilio* (Poepp. & Endl.) Krasser			X
Onagraceae	*Oenothera magellanica* Phil.			X
Iridaceae	*Olsynium biflorum* (Thunb.) Goldblatt			X
Iridaceae	*Olsynium frigidum* (Poepp.) Goldblatt	X		
Iridaceae	*Olsynium junceum* (E. Mey. ex C. Presl) Goldblatt	X		
Iridaceae	*Olsynium scirpoideum* (Poepp.) Goldblatt	X	X	
Brassicaceae	*Onuris papillosa* O.E. Schulz			X
Rubiaceae	*Oreopolus glacialis* (Poepp.) Ricardi	X		X
Asteraceae	*Oriastrum lycopodioides* (J. Remy) Wedd.		X	
Asteraceae	*Oriastrum pusillum* Poepp. & Endl.		X	X
Apiaceae	*Osmorhiza chilensis* Hook. & Arn.	X		
Oxalidaceae	*Oxalis adenophylla* Gillies ex Hook. & Arn.		X	
Oxalidaceae	*Oxalis compacta* Gillies ex Hook. & Arn.	X		
Oxalidaceae	*Oxalis enneaphylla* Cav.			X
Oxalidaceae	*Oxalis laciniata* Cav.			X
Oxalidaceae	*Oxalis penicillata* Phil.	X		
Oxalidaceae	*Oxalis squamata* Zucc.	X		
Poaceae	*Pappostipa chrysophylla* (E. Desv.) Romasch.		X	X
Asteraceae	*Perezia carthamoides* (D. Don) Hook. & Arn.	X		
Asteraceae	*Perezia linearis* Less.			X
Asteraceae	*Perezia lyrata* (J. Remy) Wedd.		X	
Asteraceae	*Perezia pilifera* (D. Don) Hook. & Arn.	X		X
Ericaceae	*Pernettya prostrata* (Cav.) DC.			X
Caryophyllaceae	*Petrorhagia dubia* (Raf.) G. López & Romo			X
Boraginaceae	*Phacelia secunda* J.F. Gmel.	X	X	X
Poaceae	*Phleum alpinum* L.			
Plantaginaceae	*Plantago hispidula* Ruiz & Pav.		X	
Plantaginaceae	*Plantago lanceolata* L.			X
Poaceae	*Poa alopecurus* (Gaudich. ex Mirb.) Kunth			X

Poaceae	*Poa* gr. *denudata* 1	X	X	
Poaceae	*Poa* gr. *denudata* 2	X	X	
Poaceae	*Poa* gr. *denudata* 3	X		
Poaceae	*Poa lanuginosa* Poir. (Phil.) Giussani & Soreng			X
Poaceae	*Poa spiciformis* (Steud.) Hauman & Parodi			X
Polygalaceae	*Polygala salasiana* Gay		X	
Apiaceae	*Pozoa coriacea* Lag.	X	X	
Schoepfiaceae	*Quinchamalium chilense* Molina	X	X	
Liliaceae	*Ribes magellanicum* Poir.			X
Polygonaceae	*Rumex acetosell*a L.			X
Poaceae	*Rytidosperma lechleri* Steud.	X		
Poaceae	*Rytidosperma pictum* (Nees & Meyen) Nicora	X		
Poaceae	*Rytidosperma virescens* (E. Desv.) Nicora	X	X	X
Apiaceae	*Sanicula graveolens* Poepp. ex DC.	X	X	
Saxifragaceae	*Saxifraga magellanica* Poir.			X
Lamiaceae	*Scutellaria nummulariifolia* Hook. f.			X
Asteraceae	*Senecio alloeophyllu*s O. Hoffm.			X
Asteraceae	*Senecio argyreus* Phil.			X
Asteraceae	*Senecio clarioneifolius* J. Rémy	X		
Asteraceae	*Senecio crithmoides* Hook. & Arn.		X	
Asteraceae	*Senecio francisci* Phil.	X		
Asteraceae	*Senecio gnidioides* Phil.			X
Asteraceae	*Senecio laseguei* Hombr. & Jacquinot			X
Asteraceae	*Senecio looseri* Cabrera	X		
Asteraceae	*Senecio pachyphyllos* J. Remy		X	
Asteraceae	*Senecio patagonicus* Hook. & Arn.			X
Asteraceae	*Senecio peteroanus* Phil.	X	X	
Asteraceae	*Senecio* sp. 1			X
Asteraceae	*Senecio* sp. 2			X
Asteraceae	*Senecio* sp. 3			X
Asteraceae	*Senecio* sp. 4		X	
Asteraceae	*Senecio subumbellatus* Phil.		X	
Asteraceae	*Senecio vaginifolius* Sch. Bip.			X
Caryophyllaceae	*Silene chilensis* (Naudin) Bocquet		X	
Caryophyllaceae	*Silene magellanica* (Desr.) Bocquet			X
Iridaceae	*Sisyrinchium arenarium* Poepp.	X	X	X
Iridaceae	*Solenomelus segethii (*Phil.) Kuntze	X	X	
Lamiaceae	*Stachys grandidentata* Lindl.	X		
Asteraceae	*Taraxacum gilliesii* Hook. & Arn.			X
Asteraceae	*Taraxacum officinale* G. Weber ex F.H. Wigg.	X		
Poaceae	*Trisetum preslei* (Kunth) E. Desv.	X	X	
Poaceae	*Trisetum* sp.			X
Poaceae	*Trisetum spicatum* (L.) K. Richt.	X	X	X
Liliaceae	*Tristagma nivale* Poepp. f.			X
Asteraceae	*Tristaria* sp.		X	
Tropaeolaceae	*Tropaeolum polyphyllum* Cav.	X		

Valerianaceae	*Valeriana carnosa* Sm.			
Valerianaceae	*Valeriana philippiana* Briq.			X
Fabaceae	*Vicia bijuga* Gillies ex Hook. & Arn.		X	X
Violaceae	*Viola atropurpurea* Leyb.	X		
Violaceae	*Viola cotyledon* Ging.		X	
Violaceae	*Viola maculata* Cav.			X
Violaceae	*Viola philippii* Leyb.	X		
Liliaceae	*Zoellnerallium andinum* (Poepp.)	X		
