# Supplementary material for: Determinants of high mountain plant diversity in the Chilean Andes: From regional to local spatial scales
Source: PLoS One. 2018 Jul 6;13(7):e0200216. doi: 10.1371/journal.pone.0200216 (PMC6034847; doi:10.1371/journal.pone.0200216)
Supplement: S2 Table — (PDF) [file pone.0200216.s002.pdf]

S2 Table: The mean (± standard error) of all diversity metrics on three regions at plot, quadrat and cell scales.

Region	Species richness			Inverse of Simpson's concentration index			Beta-diversity			Plant cover		
plot scale												
Farellones	20.82	±	1.69	8.97	±	1.23	0.82	±	0.02	20.00	±	2.35
Maule	22.33	±	2.67	11.80	±	1.73	0.87	±	0.02	14.51	±	2.64
Torres del Paine	29.80	±	2.80	15.69	±	1.75	0.72	±	0.02	29.79	±	4.56
quadrat scale												
Farellones	11.05	±	0.58	5.86	±	0.37	0.47	±	0.15	20.00	±	13.01
Maule	11.78	±	0.71	7.25	±	0.47	0.47	±	0.1	14.51	±	13.45
Torres del Paine	16.94	±	1.01	9.40	±	0.62	0.52	±	0.13	32.17	±	32.19
cell scale												
Farellones	2.37	±	0.07	1.92	±	0.05	0.75	±	0.16	27.3	±	27.52
Maule	2.40	±	0.08	1.82	±	0.06	0.82	±	0.15	16.07	±	23.06
Torres del Paine	4.17	±	0.12	2.74	±	0.08	0.80	±	0.18	19.14	±	27.93
